# Past Fame, Present Frames and Future Flagship? An Exploration of How Health is Positioned in Canadian Foreign Policy

**DOI:** 10.3390/admsci2020162

**Published:** 2012-06-01

**Authors:** Ronald Labonté, Vivien Runnels, Michelle Gagnon

**Affiliations:** 1 Globalization and Health Equity, Institute of Population Health, University of Ottawa, 1 Stewart Street, Ottawa, ON K1N 6N5, Canada; 2 Department of Epidemiology and Community Medicine, University of Ottawa, 1 Stewart Street, Ottawa, ON K1N 6N5, Canada; 3 Institute of Population Health, University of Ottawa, 1 Stewart Street, Ottawa, ON K1N 6N5, Canada

**Keywords:** global health, foreign policy, Canada, security, trade, development, human rights, global health diplomacy

## Abstract

Canada has been regarded as a model global citizen with firm commitments to multilateralism. It has also played important roles in several international health treaties and conventions in recent years. There are now concerns that its interests in health as a foreign policy goal may be diminishing. This article reports on a thematic analysis of key Canadian foreign policy statements issued over the past decade, and interviews with key informants knowledgeable of, or experienced in the interstices of Canadian health and foreign policy. It finds that health is primarily and increasingly framed in relation to national security and economic interests. Little attention has been given to human rights obligations relevant to health as a foreign policy issue, and global health is not seen as a priority of the present government. Global health is nonetheless regarded as something with which Canadian foreign policy must engage, if only because of Canada’s membership in many United Nations and other multilateral fora. Development of a single global health strategy or framework is seen as important to improve intersectoral cooperation on health issues, and foreign policy coherence. There remains a cautious optimism that health could become the base from which Canada reasserts its internationalist status.

## 1. Introduction

In 2007, the foreign ministers of seven countries (Norway, France, Brazil, Indonesia, Senegal, South Africa and Thailand) issued the Oslo Declaration identifying global health as ‘a pressing foreign policy issue of our time’ [1]. A number of government legislative, policy or commentary reports preceded and followed: Sweden’s policy on development [2], Switzerland’s health foreign policy [3], Norway’s Policy Coherence Commission [4] and new white paper on global health in foreign and development policy [5–7], and the UK’s Health is Global strategy [8]. In 2009, the US Institute of Medicine issued the second of two reports on global health in foreign policy [9], the same year that the Annual Ministerial Review held by the UN Economic and Social Council devoted itself to global public health. In November 2008 fifty-five nations sponsored a UN General Assembly Resolution on global health and foreign policy, urging member states ‘to consider health issues in the formulation of foreign policy’ [10]. Such developments serve to demonstrate a decadal trend in which health has risen to become an integral part of global policy discourse, accompanied by increased global financing for health, a proliferation of new health intervention initiatives (such as the Global Fund to Fight AIDS, Tuberculosis and Malaria and the GAVI Alliance), the implementation and discussion of innovative financing and taxation schemes (such as UNITAID’s airline tax and the International Finance Facility for Immunisation), and new private players (notably the Bill and Melinda Gates Foundation).

There are concerns that 2008’s global financial crisis has shifted international policy priorities elsewhere and that the decade of health is waning [11]. The post-crisis austerity agenda has reduced the annual growth rate in global financing for health [12], and avoiding the destabilizing effects of a full-blown recession has assumed international centre stage. Yet attention to global health, if subdued, is not absent. The United Nations’ General Assembly convened only its second summit focussed on health ^1^ in September 2011, addressing the growing pandemic of non-communicable diseases [13], and several UN agencies have expressed concern with the post-crisis ‘austerity agenda’ and its negative effects on health and social protection policies in developed and developing countries alike [14–16]. Most recently, the original group of nations that issued the Oslo Declaration expressed support for a new two year commission on global governance for health being led by *The Lancet* and two universities [17,18]. Additionally, the US Department of Health and Human Services released its new global health strategy in October 2011 [19].

Canada, until recently regarded as a pioneer in health multilateralism through its demonstration of leadership in the Ottawa Agreement to ban the use of anti-personnel landmines and in negotiations over the Framework Convention on Tobacco Control [20], has yet to produce any formal global health policy or framework. The lack of an explicit governmental policy does not necessarily mean that interest in the intersection of global health with foreign policy is absent. In 2010, for example, Health Canada commissioned two policy reviews to examine the bases for global health as a foreign policy goal [21,22]. Further, two research projects were undertaken around the same time examining global health diplomacy in Canada, one by Hoffman [23], and the study reported in this article. Most recently, the Canadian Academy of Health Sciences (CAHS) completed an expert panel review of Canada’s strategic role in global health [20]. These scholarly activities provide a base from which to examine the role of global health in Canada’s recent past, present and near-future foreign policy engagements.

While this paper focuses on Canada, its findings contribute to a still relatively sparse but growing body of literature focused on understanding how and why health is integrated into foreign policy. Key questions generated from this study for further exploration include how to assess the impact of national global health strategies on global health practice and outcomes and the factors that appear to align with effective global health leadership at the country level, including, strong academic and civil society engagement, political will and leadership within the bureaucracy.

## 2. Canadian Case Study

Our study set out to examine how global health has been considered in Canadian foreign policy. We were further interested in how global health is understood by Canadian foreign policy makers; is the perspective one that is narrowly focused only on disease and health/medical care (including drug research or access to essential medicines), or does it incorporate a broad understanding of the importance of social determinants of health? Relatedly, we wanted to explore which global health issues had most policy traction, which state or non-state actors are involved in framing health as a foreign policy goal, and what have been enabling or constraining factors in positioning health (and health equity) more prominently in Canadian foreign policy deliberations. In this article we focus on how health has been framed or defined as a foreign policy concern.

## 3. Research Methods

Two methods were employed in our study. The first method involved a systematic document review and analysis of recent (post-2000) Canadian federal government policy statements on global or international health, or other policies and statements issued by federal Ministries or departments whose activities have strong if indirect bearing on health. Ten such documents were located (Table 1). A discourse analysis of these documents was conducted searching for specific references to how health was framed as a policy issue. An initial template of policy framings, based upon earlier work by the researchers [24,25] was used, followed by a thematic coding to ensure that novel rationales were also captured.

The second method was key informant interviews using purposive and snowball sampling. Thirteen (13) interviews were conducted with persons from the government, academic research and civil society sectors. All interviewees were recruited on the basis of their recent and current engagement in global health and foreign policy. Interviews took place over a 6 month period in mid-2011 and were conducted by phone or in person. Each interview lasted from 45 to 90 minutes, and was audio-taped, transcribed and checked for accuracy. Transcripts were returned to interviewees for member-checking if they so requested. Interview data were thematically coded with the assistance of qualitative data analysis software, NVivo. Our interview sample appears to differ from that used in the study by Hoffman [23], which preceded ours by less than a year and recruited only senior public servants. While we included some senior public servants in our sample, none referred to their participation in the earlier study by Hoffman from which we presume that there is little if any overlap in interviewees. Many of the findings in both studies are similar and will be noted as such in our analysis. This triangulation across samples indicates where inferences drawn from the interview data can be considered to be more robust.

The study was approved by the University of Ottawa Research Ethics Board.

## 4. Situating the Case

As in most countries, there are multiple actors involved in setting foreign policy agendas. The major actors in Canada at the time of our study were the Department of Foreign Affairs and International Trade (which briefly had within it a small group devoted to health issues), the International Affairs Directorate of Health Canada (which coordinates Canada’s representation at the World Health Organization and other multilateral bodies, and provides health input when requested on trade, investment and other foreign policy issues), the Public Health Agency of Canada (which is the lead group for intergovernmental pandemic preparedness and risk management policies related to other cross-border health threats) and the Canadian International Development Agency (CIDA), the country’s lead development agency. Other groups include the International Development Research Centre, a public corporation that supports research in low- and middle-income countries to improve social, economic, environmental and health conditions; the Canadian Academy of Health Sciences, comprised of academic health scientists with the objective of providing independent assessments of issues important to the health of Canadians including global health; and a number of non-governmental organizations involved in global health and development programs. Relations between these actors respecting health and foreign policy can be strained at times, as some of the findings (below) discuss.

## 5. Findings

Our findings are described under the following thematic categories:

- Why should Canada care about global health?
- Positioning health in Canadian foreign policy
  - Security
  - Trade
  - Development
  - Human rights
- Whither health in Canadian foreign policy?
- Building a coherent global health strategy for Canada

Direct quotes from those who were interviewed are written in italics.

### 5.1. Why Should Canada Care about Global Health?

There was a strong perception amongst our informants that health has become an unavoidable element in Canadian foreign policy and hence diplomacy, and for at least four reasons. First, it is part of the routine multilateralism of Canada’s international obligations (such as those concerning the World Health Organization (WHO) or the Pan-American Health Organization):

The government is forced to engage on health, in negotiations on health, regardless of whether it wants to or not, simply because these topics emerge within discussions at international venues.

Second, negotiations in sectors other than health are increasingly raising health implications:

Environment, human rights, development work across the board and trade as well … more and more are starting to have very strong health components in them.

Canada’s diplomats involved in these sectors increasingly are “*going to be encountering health issues and [may] not be well-equipped to understand [them];*” while pragmatically, “*global health issues are not going to go away*” and Canada needs to anticipate better “*some of the global health challenges that might be coming down the pipe*.” Third, the health effects of these challenges increasingly cross borders: there is a general recognition of the inherently global nature of many of today’s domestically experienced health problems, “*all of the stuff about globalization.*” Trade, migration flows, shifting disease burdens, inequitable allocation of resources for health, financial crises and even the fear of unchecked diseases de-stabilizing countries or regions have all been proffered as reasons why Canada should care about global health [20,23,25]. The fourth reason is simply international peer pressure.

The ‘everybody’s doing it argument’: when the [federal] Ministry contests what you’re doing you [say] ‘Hey everyone else is doing it so why shouldn’t we?’

Several of Hoffman’s respondents believed that Canada was already ‘doing it,’ that the country was “a strong leader in global health affairs” [23]. Our own findings were less sanguine on this account:

Canada is definitely not seeing the value of health and the opportunities presented by health engagement…and I think it’s to our detriment.

[T]here is a presumption… that global health is of interest to Canada’s foreign public policy, and I would say, is it? For the last of at least eight years I can’t remember a document that would clearly say that health is a global foreign affairs goal of Canada …

Although there was a sense that many of Canada’s recent statements bearing on global health were being ignored or were simply *“absent from the policy scene,”* these statements still define the work of many of those we interviewed. The ability to position health more prominently in foreign policy depends on the justifications or arguments advanced for its consideration. Our study was particularly interested in understanding how Canadian policy statements and those involved in global health diplomacy saw health as a foreign policy goal.

### 5.2. Positioning Health in Canadian Foreign Policy

Several arguments, or policy frames, for health in foreign policy have been advanced in recent years. We used a template of six in our policy analyses, and as a guideline for our interviews and analyses: health as an issue of security, trade, development, global public good, human rights and ethical/moral imperative. These policy frames are similar to those found in the UK’s global health strategy [8], are referenced in Hoffman’s 2010 Canadian study [23] and overlap with those identified by other international health scholars [26]. Reference to all six of these global health frames can be found in one or more of the policy documents we reviewed for this study, and in how many of our key informants described health as a Canadian foreign policy goal. However, only security, trade, development and human rights received more than cursory mention, and thus are the foci of the discussion that follows.

#### 5.2.1. Security

The security frame predominates, not only in Canada but in the global health policy statements of many countries [25]. This is unsurprising given that security is considered one of the ‘high politics’ in international relations theory, the other being economic advantage in which international trade figures prominently. Two of Canada’s recent foreign policy texts, its National Security Policy [27] and International Policy Statement [28], cite security threats, terrorism and the risk of failed or fragile states as the context for a new approach to foreign policy, similar to arguments advanced a few years earlier in the USA [29] and repeated in its 2011 global health strategy [19]. The National Security Policy identifies three core interests: protecting Canada and Canadian citizens at home and abroad, ensuring Canada does not harbour security threats to its allies and contributing to international security measures. Several of our informants reflected this concern:

Canadians themselves need protection both at home and abroad. So some of the work that we’re actually doing outside of Canada and some of the changes that we can help influence through foreign policy changes should be able to benefit Canadians when they travel abroad. That’s especially true … for things such as infectious diseases … but also other security risks … including war…

One of our informants further argued that curbing disease prevalence in other countries has indirect gains beyond simply health protection for Canadians, hinting at the benefits of preventing disease-induced economic decline as well:

If we can actually help influence through foreign policy, changes in other countries that yield [improvements in] both communicable and non-communicable diseases, then we are also helping ourselves by helping them. [W]e will be the recipients of either some of the products [they trade] or some of the individuals [who migrate] who will benefit [us] as a result … So it’s a concept of … enlightened self- interest.

Pandemic fear remains the most important motivation for countries to develop or amend their global health strategies. Put simply by one of our informants, *“diseases don’t really have boundaries”.* The risk of avian flu and other pandemics, such as MDR and XDR tuberculosis which now comprise up to 2 per cent of all Canadian cases [30], led to new interdepartmental coordination across the federal Canadian government; but “there is not the same kind of mechanisms for people to meet and discuss general global health issues” [23]. As one of our informants expressed:

When SARS happened, you could bring global health, be able to talk about global health in a foreign policy context because it was clearly of interest not just to Canada but to the rest of the world.

However, extending global health interest beyond pandemic risk was seen as constrained, rather than enabled, by an emphasis on state security:

A state security perspective—it’s really rooted in protection of [the] self and of one’s interests. It’s not rooted in a conception that recognizes … in the global sense that the health of one depends on the health of the other. TB … and HIV are two very good examples of that.

The ‘global sense’ to which this informant refers aligns more closely with human rather than national security. Referenced briefly in the International Policy Statement [28], the concept of human security has Canadian origins in its championing by a former Foreign Affairs Minister, Lloyd Axworthy, during his 1996–2000 tenure. Unlike national security, it is people- rather than state-centred with particular emphasis placed on vulnerable populations. Human security is cited as one of the principles for a new Canadian global health strategy in the CAHS report [20], and implies a concern for global health that transcends narrow national interests. There is no reference to human security in recent government statements, and there has been little change in Canada’s security framework since 2004 apart from a focus on building domestic capacities (‘critical infrastructure’) to strengthen the country’s response to security risks [31].

Canada’s most recent policy statements are fairly unequivocal about its national and economic self-interests in foreign affairs. The 2009/10 Priorities for the Department of Foreign Affairs and International Trade [32] emphasize closer ties with the Americas (notably the USA) for purposes of security, the war in Afghanistan and increasing Canada’s global competitiveness. Health is seen only as a potential threat (cross-border pandemic). There is no reference to mutually beneficial global prosperity (one of the arguments advanced in the 2005 International Policy Statement), only to “advancing Canadian interests” and “Canada’s competitive position in the global economy” [32]. The most recent 2011/2012 Priorities emphasize economic competitiveness even more strongly [33]. Health is not mentioned at all, while ‘security’ occurs eight times in this one page statement. A further indication of the pre-eminence given to the security agenda was the creation of a new Cabinet Committee on National Security in May 2011 [34].

#### 5.2.2. Trade

Trade interests also figure prominently in Canadian foreign policy statements. The International Policy Statement highlighted liberalized trade as simultaneously important to Canada’s global competitiveness and to the development needs of poorer countries, citing a convergence with national security interests:

Through Doha [a reference to the current round of World Trade Organization negotiations], developed states stand to gain a more liberal trading regime while developing countries will get a fairer deal. Here, as in the security realm, national interests can be brought to converge with common interests [28].^2^

This statement is similar to a conventional economic argument that trade liberalization increases growth and development, which reduces poverty, which leads to better health that in turn improves growth. This suggests that more open markets create aggregate global welfare gains for all countries, including Canada, repeating an argument made in Canada’s policy statement on the WTO in 2000 [35]:

The open world trading system that we have collectively constructed over the past 50 years has provided tremendous benefits to all WTO Members, and especially to those members, like Canada, that have opened themselves to the world. The evidence is clear—trade creates better jobs, increases access to goods, services, and technology at competitive prices, and generates revenues to support social programs. An open, healthy trading system is critical to Canada’s continued prosperity and the economic and social well-being of all nations.

No references for these claims are cited and, while compelling, the argument lacks strong empirical evidence or, at best, is highly contested [36–39]. Canada’s recent stance on international trade even more clearly prioritizes Canadian economic gains through increased market access in developing countries, and advancing Canadian interests in the Doha Round [40]. (The Round started on the assumption of ensuring disproportionate gains for developing countries, of which Canada is not one.)

Trade-related arguments for health cohere with foreign policy’s high politics and, as one informant bluntly stated in explaining how political support for global health might be gained: “*economic arguments would likely be most successful.*” This ‘business case’ for global health investment, effectively consolidated in the 2001 WHO Commission on Macroeconomics and Health [41], is still dominant in global health discourse, with the clearest Canadian statement on the instrumental argument for global health investments coming from its 2007 International Health Strategies report:

We view health as a fundamental investment which has both economic and political benefits. Healthy people are productive people who make important contributions to the economic well-being of their country [42].

As another of our informants expressed:

The economic argument about productivity is something that can’t be refuted. You know, people are sick, they don’t have energy because they’ve got malaria, diarrhea, they don’t show up for work, they’re not productive; it affects the productivity of a country. It stuns people that it’s also the truth. You can say, oh … in general it’s a good thing to be healthy, and it is. But from an economic point of view, I think that’s a critical piece.

This passage implies that Canada’s own trade interests have some dependence upon the health and economic capacities of developing countries, either as producers of goods it imports or as purchasers of goods and services it exports. As one of our informants summarized:

Rather than trade issues trumping health I think actually health and global health issues can facilitate better trade, smarter trade and more trade. If you can start to demonstrate that Canadian products actually have achieved a certain level of either efficacy or safety or benefit, from a health perspective, you can actually use that to help facilitate trade.

This health and trade alignment speaks more to Canada’s own economic advantages than to any normative or ethical imperative to contribute to greater global health security. As another of our informants sharply complained:

The trade agenda and the economic prosperity agenda have surpassed and supplanted some of the other reasons why we do things internationally … Really it’s primarily about trade, and the social and the cultural and the health issues tend to play second or third or fourth fiddle when foreign policies are being discussed.

Trade and mercantilist sentiments also defined Canada’s 2007 Science and Technology Strategy [43]. This strategy called for harnessing scientific research to private sector entrepreneurialism noting that “the most important role of the Government of Canada is to ensure a competitive marketplace and create an investment climate that encourages the private sector to compete against the world on the basis of their innovative products, services and technologies” [43]. Health is one of the areas identified in this strategy, under a principle of “discovery and commercialization in Canada.” Such research, while having “an economic benefit to Canada,” is also argued to help “limit the spread of diseases and potential pandemics” and reduce global poverty. The CAHS report makes a similar claim, but further cautions that most health technology research is aimed at markets in wealthier countries, and that when new drugs or therapies are discovered they are often “prohibitively expensive” for most people living in poorer countries [20].

Canada was the first country to pass legislation to permit the production and export of generic medicines to developing countries lacking production facilities, following belaboured reform of the TRIPS agreement in 2003. Its legislation, positively regarded by several of our informants as a global ‘first’, proved to be unduly cumbersome and in three years led to only one compulsory license for two shipments of HIV medicines to Rwanda. A new Bill to dramatically streamline the process was passed by Canada’s federal parliament in early March 2011, but was stalled by Canada’s Senate (deliberately, according to several civil society organizations) and died on the order paper when a federal election was called later that month [44]. Whether the Bill will be re-introduced is unknown; but as one of our informants commented on how Canada now positions itself in multilateral meetings discussing access to essential medicines:

…some countries will align themselves more to producers of generic drugs, for example India, Brazil, other countries, on specific issues related to generics. Other countries will align themselves on issues related to trade, for example the United States, a lot of the developed countries including Canada.

#### 5.2.3. Development

Evidence of trade’s dominance extends to development assistance, where there has been a shift in aid orientation away from social infrastructure to trade treaty-related compliance embodying the ‘trade not aid’ assumption that increased integration into the global economy is essential for growth and development in poorer countries. As the International Policy Statement stated:

Canada can also help developing countries make the adjustments necessary to benefit from the opportunities offered by free trade. Continued support, both through development assistance and technical capacity building, are necessary if all states are to participate as equal members in the global economy [28].

The economic burdens many smaller nations face in becoming trade-treaty compliant are high and certainly do require some assistance; but there is also concern that ‘aid for trade’ is crowding out transfers for health and social investments [45] and, as previously noted, there is considerable empirical scepticism over the role global trade plays in reducing poverty or improving health in low-income countries.

Notwithstanding the incursion of trade into aid, it is the policy frame of development within which health holds its global prominence. Health has long been one of the desired outcomes of development, to which the Millennium Development Goals (MDGs) have become the normative backdrop. Two paragraphs of the Millennium Declaration, to which Canada along with most of the world’s nations have committed, state or imply a foreign policy goal of ‘generous’ wealth transfers to poorer countries:

Global challenges must be managed in a way that distributes the costs and burdens fairly in accordance with basic principles of equity and social justice. Those who suffer or who benefit least deserve help from those who benefit most [46 ¶ I.7].

We call on the industrialized countries…To grant more generous development assistance, especially to countries that are genuinely making an effort to apply their resources to poverty reduction [46 ¶ III.15].

It was slow progress towards the MDGs, particularly maternal and child health, that led to the Oslo Declaration in the first place, which advocated that donors should “push development cooperation models that match domestic commitment and reflect the requirements of those in need and not one that is characterised by charity and donors’ national interests” [1]. Evidence, however, suggests that most aid still tends to be allocated by donors’ strategic security or economic interests rather than by need [47–49].

Canada, in its International Policy Statement accepted the need to “think beyond our own national borders and take responsibility for one another… put our common humanity at the centre of our agenda” [28]. Health, along with assistance for economic growth, has been a priority area for Canadian aid since at least 2002. Arguing for health investments, however, has not been easy and has often required “*emphasizing the magnitude of certain global health problems”.* The importance of using strong evidence arguments was underscored by several of our informants: that diplomats needed to “*know something about the science”* of global health and disease, and “*to take a very neutral, science-based approach”* to positioning health more strongly in the development agenda. This argument has been made by health diplomats from other countries as well ^3^, and is credited to Canadian negotiators who helped to garner intergovernmental support for the Framework Convention on Tobacco Control (FCTC) [50,51]:

When we had successes like land mines or [the] Framework Convention on Tobacco … it was because the Canada Public Health Agency people were able to make the evidence so compelling.

Canada’s role in the FCTC was widely regarded by our informants as one of its most important examples of global health diplomacy, *“a very important and pivotal point in Canada’s use of global health within a foreign policy construct*.”

The money for the [FCTC] process and the impetus for the process at the beginning came mostly from Canada.

The general feeling [was] that first, Canada had a lot of experience in tobacco control, secondly that [this experience] would be relatively easy to export. And third … that international law needed to be something more than trade and needed to have health considerations … It [the FCTC] wasn’t necessarily the calculating, national self-interest sort of thing that the foreign policy theorists tend to assume governments engage in.

The FCTC, like the International Health Regulations, is often cited as an exemplary global public good by virtue of being an international health law. But there is almost no mention of the concept of global public goods (at least by name) in extant Canadian policy statements. We encountered the term only once, in the 2005 International Policy Statement with reference to alternative models of financing global health and development. Even if the idea of global public goods is indirectly assumed in policies on development and trade, the key rationale for financing such goods (that they compensate for market failures in their supply) appears to be absent from any Canadian foreign policy document.

Development priorities, however, are malleable, with their year-to-year unpredictability being a major concern for international efforts to ‘increase aid effectiveness’. Canada is one of the signatories to the ‘International Health Partnership +’, a multi-country initiative to improve predictability and harmonization of aid transfers. There is evidence that Canadian health aid is going increasingly towards government budget support, in keeping with the intent of this initiative; although at the same time Canada has refused to participate in the UNITAID airline tax program or to support recent and renewed calls for a financial transaction tax to help finance global health and development programs. In an updated Aid Effectiveness Agenda (2009), Canadian priorities also indicated a shift away from health systems and towards food security (though an important determinant of health), sustainable economic growth and “securing the future of children and youth” [52].

This reorientation did not preclude Canada’s Prime Minister as host of the 2010 G8 Summit from announcing $2.85 billion in funding for a new set of maternal/child health programs over a five year period (2010–2015). This ‘Muskoka Initiative,’ named for the venue of the G8 meeting, was regarded by many of our informants, and cited by the CAHS report, as one of Canada’s more prominent recent forays into global health. But less than half of the committed amount was new funding, the remainder being a re-packaging of existing allocations, and in the same year Canada announced a freeze on any increase in its development assistance budget over the same time period. This suggests that other potentially important health and development projects could be cut to finance the new Initiative. Canada’s overall assistance contributions stood at 0.34 percent of Gross National Income in 2010, ranking 13th amongst the 23 countries reporting to the OECD/DAC [53]. Perhaps more critical than aid quantity was the political decision to remove from the Initiative funding support for abortion services or for any organization supplying such services, demonstrating how the Canadian government reframed maternal and child health needs to fit its domestic political interests (social conservatism) to avoid any explicit reference to abortion or reproductive health services. The Initiative, and its exclusion of reference to abortion, was nonetheless seen as poorly managed. As one informant expressed:

It was an ill thought through Initiative that was generated at the political level rather than the bureaucratic level … it probably wasn’t proposed by the bureaucrats because the health advisors knew how politically contentious this would be, knew that it would touch issues such as sexual and reproductive health and abortion and knew to avoid it as a result of that.

And as another commented;

I think … it shows sort of a naïve-ness on the part of the government in terms of how international policy works, in terms of how to negotiate multi-lateral initiatives, how to approach that, how to build consensus, and also just flat out ignorance about maternal health.

On the one hand, the health system funding released under this Initiative was viewed positively:

I’d say the Canadian government’s position on this has meant that rather than concentrate on sexual and reproductive health… it’s focusing more on health system strengthening, HIV, training birth attendants, … all good work..

But on the other hand:

If there’s conditionality that you cannot provide family planning or safe abortion services as part of the work that you’re doing, that’s going to hamper a lot of work.

#### 5.2.4. Human Rights

The controversy over the Muskoka Initiative draws the issue of health and foreign policy into the terrain of human rights. Article 12 (the ‘right to health’) of the International Covenant on Economic, Social and Cultural Rights (ICESCR) includes within it state obligations to ensure access to reproductive health services [54]. Although no mention is made of abortion, lack of access to safe abortion services is considered by some rights-based organizations to constitute a violation of women’s right to health [55].

The need and obligation to respect human rights, democracy and the rule of law underpinned Canada’s International Policy Statement [28] and is included as an important component of Canada’s strategy with the Americas in the 2009–2010 Department of Foreign Affairs and International Trade’s (DFAIT) priorities [32]. Several of our informants thought that a rights-based approach was important in advancing Canadian actions in global health, notably maternal health (bracketing the issue of abortion):

Moving into the human rights box really puts the focus on the women who experience death due to pregnancy or childbirth … and you’re also focusing it on government obligations. Some governments … don’t want to necessarily acknowledge that they have human rights obligations with respect to this … and that these deaths actually represent a failure on their part to fulfill their obligations under the right to health and other human rights.

Similarly, with respect to Canada’s role to provide humanitarian assistance:

We have a duty, and this isn’t mentioned very often, you really don’t hear it much anymore, about Canada’s… obligations. It’s often relegated if at all to a fairly sort of minor role as a justification for engagement on the global stage, but I’d put it forward as there is an obligation on us to provide assistance and to help build capacity and to help improve the issue of equity in access [to health].

The sentiments expressed by these informants are consistent with expert opinions of state obligations under the ICESCR [56] and other international human rights and humanitarian law covenants [57]. Canada has ratified both the ICESCR and the International Covenant on Civil and Political Rights, including the optional protocol allowing individuals to bring complaints before the UN Commission on Human Rights, widely regarded as a progressive development in international human rights jurisprudence. Although there is generic reference to human rights in some of Canada’s recent policy statements, there is no specific reference to human rights obligations under international covenants, resulting in considerable scepticism amongst our informants of how persuasive such arguments have been or will be in shaping Canadian foreign policy:

Generally with human rights matters I don’t think you see a lot of discussion within the government itself on these human rights obligations, on new international policy in the area of human rights; I don’t think you see a lot of discussion regardless of the actual government.

### 5.3. Whither Health in Canadian Foreign Policy?

International relations scholars argue that national security and economic interests will often conflict with and inevitably trump that of global development and humanitarian aid, the domains within which most global health issues reside. Our study’s findings offer little evidence to challenge the supposition that health, when it does rise in Canadian foreign policy, does so primarily for instrumental reasons. As one informant stated:

[Federal Canadian] departments first and foremost have a responsibility to do things for the national interest [and] engagement on the global health scene [is of] secondary interest. Often you really have to justify why it is that you’re actually engaged in a health issue outside of your country … it’s difficult [and] it’s something that you’re constantly being challenged on …

Narrowly construed domestic interests will often over-ride those of longer term global health need [58], or, what does not affect us, does not concern us, a point made by another of our informants:

It’s always easier to gain political support for a genuine domestic problem, however marginalized the population that’s affected by that problem is. And I think that’s really the fundamental challenge … There are no Canadians who get Leishmaniasis or African sleeping sickness.

For some of our informants, this was simply a given of politics:

No politician builds their career or their electoral campaign … based on a global health or global health and foreign policy. The politician who becomes personally involved is an extreme exception.

Exceptions do arise, as they have in the UK, Switzerland, Norway and Brazil, where health in foreign policy is more prominent. The value of career civil servants to global health diplomacy was also emphasized by several of our informants:

Politicians may raise [global health] issues but they aren’t always there for the whole process … Most of [the work] is carried [out] by bureaucrats.

In carrying out this work the issue of mandates arises. Hoffman’s study found that domestic mandates hobbled the international efforts of what he called the ‘Health Portfolio’ of Health Canada and the Public Health Agency of Canada. Both of these federal departments have international affairs directorates but are constrained unless they can “justify all [their] international activities as benefiting the health of Canadians” [23]. Their global health remits appear limited to the health multilaterals (the World Health Organization, the Pan-American Health Organization) and to specific disease-related concerns, such as pandemic preparedness and, more recently, multilateral initiatives on control of non-communicable diseases.

As one of our informants explained, “*health in [foreign policy] is only seen as a development issue.*” Within the Canadian federal panoply, development falls under Canada’s international aid program, administered by the Canadian International Development Agency (CIDA), “*the primary funding agency to actually place that link between health and foreign policy.*” Those with a health, rather than development, mandate found the health leadership role assigned to CIDA problematic:

You have CIDA spending money on global health but Health Canada not having any money or not having any input. You have CIDA articulating things from a developmental perspective which might not necessarily be in the public health interest of Canadians.

At the same time, as others noted:

Often the people within a sectoral department like Health Canada or PHAC don’t have the experience in foreign affairs or in development assistance to be able to see how the connections are made, and vice versa.

Fundamentally, however, and as one exasperated veteran in global health lamented:

There’s a real lack of institutional cohesion in global health amongst CIDA, Health Canada and the Public Health Agency. There’s no global health strategy. If you were to ask what [are] Canada’s objectives are in global health … you’d probably get 10 different answers.

Both Hoffman’s and our own study found mixed opinions on how well collaboration across Canada’s federal departments was occurring. Although the intent by many policy workers was there, it was constrained by the usual silos affecting competition over voice and resources. Moreover, as another of Hoffman’s informants noted, “To have a coherent approach to global health, you need a strategic vision, goals and direction” [23]. There are “tons of working groups” and “networks” but no single cluster with a mandate to forge a Canadian global health strategy or see that it is represented in all Canadian foreign policy discussions. As one of our informants commented:

There used to be a [health] team [in foreign affairs]. And a really very bright group of people who were not only really good in terms of substantive [issues] but they were very progressive and very good at going to other governments and influencing their positions. And other governments relied on them because they had built up this level of expertise. So there’s this whole thing about the loss of Canada’s leadership position on foreign policy.

The health team in DFAIT was disbanded as a cost-saving measure, ironically around the same time as the Muskoka Initiative was announced. For some this loss made it more difficult to position health in foreign policy:

You don’t have the group in Foreign Affairs anymore that actually deals with health, so there’s probably a lack of understanding of [health’s] importance.

For others it reflected a problem of mandate:

While the folks doing foreign policy get it [health in foreign policy], they understand it, they don’t disagree with any of this, it’s not actually in their mandate … It’s not as a government do we say, “This is an important component of achieving foreign policy objectives.” … Until we start bringing these formal links that essentially recognizes that you can’t achieve … foreign policies in many areas without also thinking of [health] … it will be hard to move forward.

### 5.4. Building a Coherent Global Health Strategy for Canada

The way to move forward was linked to the development of a single global health strategic framework:

… having people work together, a framework which … would include some resources for Health Canada and the Public Health Agency to work internationally would help in terms of efficiency and the policy coherence within the Canadian government.

A 2011 Stakeholder Forum on “Health and Emerging Global Issues in Canada” convened by McMaster University in Ontario similarly “embraced the idea of providing a framework for government and stakeholder action and coordinating government action” [59]. Participants noted that a framework must articulate the security, economic, moral and other principles or imperatives that drive a commitment to global issues (each of which speak in different ways to different communities) and adopt a health lens to examine these issues [59].

Our informants believed that a framework was important not only as a means to improve and rationalize resources for global health but also as a timely and important political gesture:

An umbrella, a good show of a political will and direction and interest … And if resources follow then an appropriate structure will be established to respond to that. Because you don’t have that kind of a statement and resources [and] you have pieces in different departments doing things in their own way.

Other countries’ global health strategies were viewed as positive examples for Canada to follow:

The UK and Norway have come up with some excellent [frameworks] … it wasn’t easy to get there and there’s still a lot of work to do with respect to implementing it in the way that they’d like to. But it’s definitely a huge step in the right direction. And it’s driven in some cases by the foreign policy people and then in some cases by their health [people].

Whether Canada follows suit remains an open question, with several informants concerned that the present government is not terribly interested in global health or that any efforts in global health diplomacy are uninformed and clumsy:

People are asking, what the hell’s wrong with the Canadian government on this [the asbestos issue] … on the mining stuff [oversight of Canadian mining operations in other countries]. I think [there’s], a belief that somehow, the health of other people in the world is actually not of importance to the Canadian government.

The reality is that in recent years the Canadian government in health diplomacy as far as I can tell has just basically been shooting itself in the foot.

Canada’s global health concerns are seen as driven very much by domestic, rather than international, interests:

There’s still a functioning health diplomacy if you like that’s trying to achieve a domestic aim for Canada, which is keeping its regulations that the Prime Minister has personally endorsed in place [such as] not losing a trade challenge internationally. But that doesn’t extend to having diplomacy about health for people outside of Canada, that’s about diplomacy for health for people in Canada.

At the same time, there was cautious optimism:

We might not be able to get the kind of global health engagement from this government that we want. But because … global health is becoming embedded in the international processes like the World Health Assembly, and you’ve got UNAIDS, you’ve got GAVI, you’ve got the Global Fund, and we’re part of that … Unless we want to withdraw [from these] we have to at least have a minimal amount of involvement.

One informant reflected on the importance Canadians themselves attach to the country’s public health system, and how this might lead to a revitalization of political interest in global health:

Canada has slipped and lost a lot of its pre-eminence globally. I think Canadians are looking for a reaffirmation of global leadership and I think health might be one of the ways to actually regain that.

## 6. Conclusion

Hoffman’s 2010 study cited a number of reasons why Canada should take its global health role more seriously: It has health systems and health research expertise, is the birth-place of evidence-based medicine, is the ‘globalization nation’ (a result of its open immigration policy giving it the highest per capita in-migration rate in the world), hosts the Global Health Security Initiative to strengthen preparedness for pandemic and other health related security threats, and “enjoys disproportionate membership in the world’s leading multilateral forums” [23]. The 2011 CAHS report, which some saw as a potential lever to create a unifying strategy, given its expert development outside of the partisan corridors of politics, builds somewhat differently upon what Canada could offer to global health [20]. It focused on Canada’s expertise in indigenous and circumpolar health, social determinants of health, community-oriented primary health care and global health education and research partnerships.

Our own study did not probe specifically for what should be the foci of a Canadian global health strategy. References, however, were variously made to human rights, pandemics, neglected diseases and maternal/child health. Even climate change was referred to in passing by several of our informants (this was before Canada singled itself out by becoming the first nation to withdraw from the Kyoto Protocol). One informant was adamant about what should be the foundation of such a strategy: “*The elements of a policy should be based on the right to health. It should be based on equity … on principles of non-discrimination. Human rights, those core set of human rights principles that inform health policy …*” Most informants, however, pointed to national security and global competitive advantage as playing the dominant roles in Canadian foreign policy: “*This idea that you have to frame [global health] in terms of power and security and national interest, maybe for this government that might be the only thing that would work …*”

The challenge is how effectively, if at all, global health can find a position within such a framing, and without losing its moral or humanitarian base. Our own study findings are inconclusive on this point; although we found a keen desire for Canada to re-assert its presence in global health. We leave the last word on that count to one of our informants:

[Health] is a win/win situation … It’s not like a trade issue, it’s not like a natural resources issue, it’s something that all countries can agree on [is] a benefit to their citizens. And I think if we can actually situate global health as a sort of a flagship of a renewed Canada foreign policy, I think that would be perceived extremely well by Canadians as being a reaffirmation of Canada’s engagement, in much the same way that we used to be considered as peace builders and peacekeepers internationally. I think Canadians are looking for that.

## Figures and Tables

**Table 1:** Canadian Policy Documents and Key Policy Statements.

Title	Year	URL
Canada’s G8 Priorities	2010	http://www.pm.gc.ca/eng/media.asp?id=3093
Canada’s International Policy Statement—A Role of Pride and Influence in the World	2005	http://publications.gc.ca/site/eng/274692/publication.html
Canada’s International Policy Statement—A Role of Pride and Influence in the World	2005	http://www.armee.gc.ca/land-terre/news-nouvelles/story-reportage-eng.asp?id=478
Department of Foreign Affairs and International Trade—Our priorities	2009–2010	http://www.international.gc.ca/about-a_propos/atip-aiprp/parl0910_atia_lai.aspx?view=d#bci_toc_listing_4
Department of Foreign Affairs and International Trade—Our priorities	2011–2012	http://www.international.gc.ca/about-a_propos/priorities-priorites.aspx?view=d
Canada’s Science and Technology Strategy	2007	http://www.ic.gc.ca/eic/site/ic1.nsf/vwapj/S&Tstrategy.pdf/$file/S&Tstrategy.pdf
Canada’s International Human Rights Policy	2010	http://www.dfait-maeci.gc.ca/rights-droits/policy-politique.aspx (2010) see also: http://www.dfait-maeci.gc.ca/department/dm_speeches/deputy-minister-speeches-2005-03-11-en.asp
Securing an Open Society: Canada’s National Security Policy	2004	http://www.pco-bcp.gc.ca/index.asp?lang=eng&page=information&sub=publications&doc=natsec-secnat/natsec-secnat_e.htm
International Health Strategies	2007	http://www.hc-sc.gc.ca/ahc-asc/activit/strateg/init-eng.php
Policy Statement on Strengthening Aid Effectiveness	2002	http://www.acdi-cida.gc.ca/acdi-cida/acdi-cida.nsf/eng/STE-32015515-SG4
Canadian International Development Agency, AID Effectiveness Agenda	2008, 2009	http://www.acdi-cida.gc.ca/acdi-cida/ACDI-CIDA.nsf/eng/FRA-1015144121-PWW

## Footnotes

1 The first summit or high level meeting on a health issues was the United Nationals Special Session on HIV/AIDS in 2001.

2 An updated version of this 2005 policy, amended in 2011, no longer contains this exact wording, although it continues to refer positively to the role of liberalized trade in achieving both security and development goals.

3 Comments made at the Global Health Diplomacy Workshop, Prince Mahidol Award Conference, Bangkok: 29–30 January 2009.
